# Prevalence of sexual problems and related help-seeking behaviors among mature adults in Brazil: data from the Global Study of Sexual Attitudes and Behaviors

**DOI:** 10.1590/S1516-31802005000500007

**Published:** 2005-09-01

**Authors:** Edson Duarte Moreira, Dale Glasser, Djanilson Barbosa dos Santos, Clive Gingell

**Keywords:** Epidemiology, Health surveys, Erec-tile dysfunction, Prevalence, Sex disorders, Epidemiologia, Levantamentos epidemiológicos, Disfunção erétil, Prevalência, Disfunção erétil, Prevalência, Sexualidade

## Abstract

**CONTEXT AND OBJECTIVE:**

Relatively little is known about the usual frequency of sexual activity and how older individuals cope with sexual problems. The objective was to study sexual activity, prevalence of sexual problems and related help-seeking behaviors among middle-aged and older men and women in Brazil.

**DESIGN AND SETTING:**

Population survey, by Fundação Oswaldo Cruz.

**METHODS:**

Interviews were held with 1,199 Brazilians aged 40-80 years (471 men and 728 women). The standardized questionnaire investigated demographics, general health, sexual behavior, attitudes and beliefs.

**RESULTS:**

Overall, 92.6% of men and 58.3% of women had had sexual intercourse during the preceding year. More than half of the men and women had done so more than once a week. Early ejaculation (30.3%) was the commonest male sexual problem, followed by inability to reach orgasm (14.0%), erectile difficulties (13.1%) and lack of sexual interest (11.2%). For women, the commonest sexual problems were lubrication difficulties (23.4%) and lack of sexual interest (22.7%). Depression was a significant correlate of sexual problems, for men and women. More women than men had sought help for sexual problem(s) from a healthcare professional.

**CONCLUSIONS:**

The findings highlight the importance of encouraging greater use of available healthcare services, including consultation with a medical doctor regarding sexual health. This should not only enable men and women to maintain satisfactory sexual function well into their later years, but may also result in overall improvement in the quality of healthcare.

## RESUMO

**CONTEXTO E OBJETIVO:**

A prevalência e os fatores correlatos de desordens sexuais masculinas como disfunção erétil e ejaculação precoce têm sido estudados em muitos países, entretanto, bem menos investigações se ocuparam especificamente dos problemas sexuais femininos. Além disso, relativamente pouco se sabe sobre a freqüência usual da atividade sexual e sobre como indivíduos mais velhos tentam lidar com seus problemas sexuais. O objetivo deste estudo foi estudar a atividade sexual, a prevalência de problemas sexuais e os comportamentos de busca de ajuda relacionados a esses problemas, entre homens e mulheres de meia-idade e mais velhos no Brasil.

**TIPO DE ESTUDO:**

Inquérito populacional realizado pela Faculdade Oswaldo Cruz. Pesquisa por telefone (discagem aleatória) conduzida no Brasil em 2001 e 2002.

**MÉTODOS:**

As entrevistas foram baseadas num questionário padronizado, incluindo informações demográficas, saúde em geral, e comportamentos, atitudes e crenças sexuais. Um total de 1.199 indivíduos no Brasil (471 homens e 728 mulheres) de 40 a 80 anos completou o inquérito.

**RESULTADOS:**

Ao todo, 92,6% dos homens e 58,3% das mulheres referiram alguma atividade sexual no ano que precedeu a entrevista, e mais da metade dos homens e mulheres reportaram atividade sexual mais de uma vez uma semana. Ejaculação precoce (30,3%) foi o problema sexual masculino mais comum, seguido por incapacidade de alcançar o orgasmo (14,0%), dificuldades de ereção (13,1%) e falta de interesse sexual (11,2%). Os problemas sexuais relatados mais freqüentemente por mulheres foram dificuldades de lubrificação (23,4%) e falta de interesse sexual (22,7%). Depressão foi correlacionada significativamente com problemas sexuais nos homens e nas mulheres. Mais mulheres do que homens tinham procurado a ajuda de um profissional de saúde para seu problema(s) sexual(is).

**CONCLUSÕES:**

Os achados do GSSAB destacam a importância de encorajar o maior uso dos serviços de saúde disponíveis, incluindo consultas médicas sobre queixas ligadas à saúde sexual. Isso deverá não somente possibilitar que homens e mulheres mantenham uma função sexual satisfatória até idades mais avançadas, mas também poderá resultar numa melhora geral da qualidade da assistência à saúde.

## INTRODUCTION

The development of several convenient and effective oral treatments for male erectile dysfunction has contributed to the increasing level of interest in the sexual functioning of middle-aged and older adults that has been seen in recent years. Over the last 10 years or so, a large number of studies have investigated the prevalence of sexual problems among middle-aged and elderly people. These studies have tended to focus mainly on the populations of individual countries in Europe,^1–5^ the United States of America (USA),^6–10^ and Central and South America.^11–15^ The prevalence and correlates of the male sexual disorders of erectile dysfunction and early ejaculation have been studied most extensively, while fewer investigations have looked specifically at female sexual problems.^16,17^ Furthermore, relatively little has been reported about the usual frequency of sexual activity and the value and significance of sexual relationships for older individuals, although the few published studies in this area have concluded that sexual interest and activity persist well into older age.^18–20^

The published literature on the prevalence and correlates of sexual disorders comprises studies conducted in developed and developing countries that have employed a wide variety of study designs and definitions. This makes it difficult to conduct cross-national comparisons in a scientifically valid manner. Moreover, there are currently no studies that allow a comparison of sexual behavior or help-seeking patterns for sexual problemsacross different countries.

The Global Study of Sexual Attitudes and Behaviors (GSSAB) was a population survey of 27,500 men and women aged 40 to 80 years in 29 countries representing many world regions.^21,23^ Brazil was one of the countries within this study.

## OBJECTIVE

Here, our goal is to report data on sexual activity, the prevalence of sexual problems and related help-seeking behaviors among men and women in the Brazilian cohort of the Global Study of Sexual Attitudes and Behaviors.

## METHODS

### Type of study

Population survey.

### Setting

A computer-assisted telephone interview survey, using random-digit dialing as the sampling design, was carried out in Brazil during 2001 and 2002 (participants were sampled from the five largest cities in Brazil, namely, São Paulo, Rio de Janeiro, Salvador, Belo Horizonte, and Brasília). Respondents were randomly selected by asking for the man or woman in the household aged between 40 and 80 years of age (participants were interviewed by interviewers of the same gender).

A structured questionnaire requested information concerning general health, demographics, relationships, and sexual behavior, attitudes and beliefs. The subjects were asked if they had engaged in sexual intercourse during the previous 12 months, and the presence of sexual dysfunction was assessed by means of two sequential questions. The respondents were first asked whether they had experienced one or more of the sexual problems listed in Tables 2a and 2b for a period of at least two months during the previous year, and those who answered ‘Yes’ were then asked whether they had experienced the problem ‘occasionally’, ‘sometimes’ or ‘frequently’.

**Table 2.** Age-standardized prevalence of sexual problems by gender in Brazil, according to severity, 2001-2002 (percentage and 95% confidence interval)

	Men (n = 471)	Women (n = 728)
**Early ejaculation**	30.3 (26.0, 34.8)	
Occasional	4.6 (2.8, 7.0)	
Periodic	14.4 (11.3, 18.1)	
Frequent	11.2 (8.4, 14.6)	
**Lubrication difficulties**		23.4 (19.4, 27.7)
Occasional		3.5 (2.0, 5.8)
Periodic		13.9 (10.8, 17.6)
Frequent		5.9 (3.9, 8.6)
**Erectile difficulties**	13.1 (10.1, 16.6)	
Occasional	4.1 (2.5, 6.4)	
Periodic	6.2 (4.1, 8.9)	
Frequent	2.8 (1.4, 4.8)	
**Lack of sexual interest**	11.2 (8.4, 14.6)	22.7 (18.8, 27.0)
Occasional	2.8 (1.4, 4.8)	3.8 (2.2, 6.1)
Periodic	6.0 (3.9, 8.6)	11.8 (8.9, 15.3)
Frequent	2.5 (1.3, 4.5)	7.1 (4.8, 10.0)
**Inability to reach orgasm**	14.0 (10.9, 17.6)	22.0 (18.1, 26.2)
Occasional	4.4 (2.6, 6.7)	4.5 (2.6, 6.7)
Periodic	7.1 (4.9, 9.9)	13.0 (9.9, 16.6)
Frequent	2.5 (1.3, 4.5)	4.5 (2.6, 6.7)
**Sex not pleasurable**	7.8 (5.5, 10.7)	20.3 (16.6, 24.5)
Occasional	3.0 (1.6, 5.0)	4.0 (2.4, 6.4)
Periodic	3.2 (1.8, 5.3)	10.4 (7.7, 13.7)
Frequent	1.6 (0.6, 3.3)	5.9 (3.9, 8.6)
**Pain during sex**	4.6 (2.8, 7.0)	18.0 (14.4, 22.0)
Occasional	1.4 (0.5, 3.0)	2.1 (1.0, 4.0)
Periodic	1.8 (0.8, 3.6)	10.6 (7.9, 14.0)
Frequent	1.4 (0.5, 3.0)	5.2 (3.3, 7.8)

**Note:** based on reports from sexually active respondents (those who had had intercourse within the past year). Percentage in the first row of each panel indicates the overall prevalence of the sexual problem, defined as experiencing the problem for a period of two months or more. The difference between the overall prevalence and the sum of the three levels of severity of each sexual problem indicates the proportion that failed to specify the level of severity. All prevalence estimates are adjusted according to the age distribution of the total of sexually active men and women in this sample from Brazil.

Logistic regression was used to investigate potential factors associated with selected sexual dysfunction. In these analyses, the presence of a sexual dysfunction was coded only for those respondents who reported experiencing the problem frequently or periodically, while those who indicated that they experienced the problem only occasionally were recorded as indicating no sexual dysfunction.

The subjects who reported that they had experienced a sexual problem were asked whether they had sought help from a number of possible sources. The options included: “Talked to partner”, “Talked to a medical doctor (other than a psychiatrist)”, “Looked for information anonymously (in books/magazines or on the internet)”, “Talked to family member or friend”, “Taken prescription drugs/devices or talked to pharmacist”, “Talked to psychiatrist or psychologist or marriage counselor”, “Talked to a cleric or religious adviser”, “Called a telephone help line” and “Other – please specify”. Respondents could indicate that they had sought help from more than one source.

The subjects with sexual problems who had not consulted a physician were asked why they had not done so, and offered a list of 14 possible reasons (from which they were to check all that applied). The reasons included attitudes and beliefs regarding the sexual problem and the patient-doctor relationship. All respondents (irrespective of whether they reported any sexual problems) were also asked “During a routine office visit or consultation in the past 3 years, has your physician asked you about possible sexual difficulties without you bringing it up first?” (Yes/No) and “Do you think a doctor should routinely ask patients about their sexual function?” (Yes/No). The categorization of household income as “low”, “medium” or “high” was based on the distribution of income in Brazil according to the criteria of the Instituto Brasileiro de Geografia e Estatística.^24^

The prevalence of a specific characteristic was calculated by dividing the number of cases by the corresponding population. The denominator for the calculation of the prevalence of sexual problem was the number of sexually active people (i.e. at least one episode of intercourse during the previous 12 months). The prevalence estimates were age-standardized using the age distribution of the population of Brazil (by gender when appropriate), and are given with their confidence intervals.^25^

## RESULTS

### Characteristics of study population

Overall, 8,637 individuals were contacted, 2,127 of whom were not eligible to participate (outside of the age range). Of the 6,510 eligible individuals, 5,088 refused to participate when the survey topic was introduced, while 223 interrupted the interview. A total of 1,199 individuals (471 men and 728 women) completed the survey, thus giving a response rate of 18.4%. Table 1 presents data on selected characteristics of the study sample, standardized for the age distribution of the population of Brazil in the year 2000. A greater proportion of men (82.6%) than women (54.3%) were married or in an ongoing partnership (Table 1). More than half of the men (57.3%) and about one-third of the women (35.0%) were employed and 66.5% of men and 54.1% of women reported that they were in good or excellent general health.

**Table 1.** Selected characteristics of the study population, Brazil, 2001-2002 (percentage; age-standardized prevalences)

	Men (n = 471)	Women (n = 728)
**Age group (years)**		
40-49	29.9	30.8
50-59	32.1	29.8
60-69	22.5	21.7
70-80	15.5	17.7
**Relationship status**		
Married or ongoing partnership	82.6	54.3
Divorced/separated without sex partner	8.9	13.2
Widowed without sex partner	4.7	22.0
Single without sex partner	3.8	10.6
**Urban residential setting**	93.8	97.4
**Education**		
Primary school or less	36.3	47.1
Secondary/high school	36.5	35.0
At least some college	27.2	17.9
**Household income**		
Low	12.4	23.8
Medium	78.5	70.8
High	9.2	5.4
**Current employment status**		
Employed	57.3	35.0
Unemployed	4.9	3.2
Retired	37.8	22.9
Homemaker	0	38.9
**Religion**		
Christian/Jewish	89.4	88.7
Atheist	3.3	1.3
Other, specified	7.3	10.0
**Good to excellent general health** *	66.5	54.1
**Intercourse in the last 12 months**	92.6	58.3
**Intercourse more than once a week**	65.6	57.2

* Self-reported “good” or “excellent” general health (versus “fair” or “poor”).

Almost all of the men (92.6%) and more than half of the women (58.3%) said that they had had sexual intercourse during the 12 months preceding the interview, and 58.4% of men and 26.1% of women engaged in sexual intercourse more than once a week.

### Prevalence of sexual problems

Early ejaculation was by far the most common male sexual problem, and was reported by almost one-third (30.3%) of the sexually active men (most of who said that they experienced this problem periodically or frequently) (Table 2). An inability to reach orgasm, erectile difficulties and a lack of sexual interest were each experienced by approximately 11 to 14% of sexually active men, while a lack of pleasure in sex (7.8%) and pain during sexual intercourse (4.5%) were much less common.

Lubrication difficulties (23.4%) were the most common sexual problem reported by sexually active women in Brazil, closely followed by lack of sexual interest (22.7%) and an inability to reach orgasm (22.0%), while a lack of pleasure in sex and pain during sexual intercourse were experienced by 20.3% and 18.0% of sexually active women, respectively (Table 2). The majority of the women who reported each of these problems said that they experienced it frequently or periodically.

Physical, health, demographic and socioeconomic factors associated with three selected sexual dysfunctions in men and women are summarized in Table 3 (odds ratios from logistic regression). Older age (age 60 to 80 years compared with the referent age of 40 to 49 years) was a significant correlate of lubrication difficulties in women (odds ratio 2.10; p < 0.05). Of the various factors investigated and listed in Table 3, three medical conditions were significantly associated with an increased likelihood of one or more types of male sexual dysfunction (i.e. a sexual problem that was experienced periodically or frequently), while only one of the factors was a significant correlate of sexual dysfunction in women. A diagnosis of depression was a significant correlate of erectile difficulties (odds ratio 3.02; p < 0.01), and early ejaculation (odds ratio 1.98; p < 0.05) among men, and of a lack of sexual interest in both men (odds ratio 4.37; p < 0.001) and women (odds ratio 1.68; p < 0.05). Diagnoses of hypertension and prostate disease were significant correlates of early ejaculation (odds ratio 1.95; p < 0.05) and a lack of sexual interest (odds ratio 2.77; p < 0.05), respectively.

**Table 3.** Factors associated with sexual dysfunction by gender, Brazil, 2001-2002

	Men			Women		
	Early ejaculation	Lack of sexual interest	Erectile difficulties	Inability to reach orgasm	Lack of sexual interest	Lubrication difficulties
**Age**						
40-49	Referent	Referent	Referent	Referent	Referent	Referent
50-59	0.70 (0.40, 1.24)	1.10 (0.40, 2.98)	0.67 (0.26, 1.73)	0.69 (0.40, 1.19)	0.68 (0.40, 1.18)	0.92 (0.54, 1.58)
60-80	0.60 (0.33, 1.09)	1.56 (0.59, 4.14)	1.04 (0.42, 2.55)	0.44 (0.24, 1.13)	0.73 (0.42, 1.27)	**2.10 (1.13, 3.91)** *
**Level of physical activity**						
average and above	Referent	Referent	Referent	Referent	Referent	Referent
lower than average	1.20 (0.66, 2.16)	0.77 (0.29, 2.07)	0.90 (0.37, 2.23)	0.85 (0.46, 1.57)	1.49 (0.88, 2.50)	0.99 (0.54, 1.80)
**Smoking**						
never	Referent	Referent	Referent	Referent	Referent	Referent
currently + smoked before	1.13 (0.70, 1.84)	1.21 (0.57, 2.59)	1.37 (0.65, 2.88)	0.72 (0.45, 1.14)	0.84 (0.54, 1.31)	0.71 (0.45, 1.14)
**Education**						
primary school or less	Referent	Referent	Referent	Referent	Referent	Referent
secondary/some college	0.70 (0.43, 1.12)	0.83 (0.40, 1.76)	0.74 (0.37, 1.74)	1.06 (0.65, 1.72)	1.44 (0.90, 2.31)	1.11 (0.68, 1.82)
**Household income**						
low	Referent	Referent	Referent	Referent	Referent	Referent
medium and high	0.69 (0.37, 1.32)	0.78 (0.29, 2.10)	1.34 (0.47, 3.84)	0.88 (0.51, 1.52)	0.81 (0.48, 1.35)	1.28 (0.71, 2.32)
**Medical Conditions**						
Depression diagnosed	**1.98 (1.04, 3.78)** *	**4.37 (1.93, 9.86)** ‡	**3.02 (1.35, 6.73)** †	1.50 (0.91, 2.46)	**1.68 (1.06, 2.67)** *	1.51 (0.92, 2.50)
Hypertension diagnosed	**1.95 (1.17, 3.25)** *	0.52 (0.21, 1.26)	1.81 (0.86, 3.79)	1.55 (0.94, 2.57)	1.00 (0.61, 1.63)	0.90 (0.53, 1.53)
Diabetes diagnosed	1.13 (0.50, 2.60)	1.53 (0.50, 4.73)	2.15 (0.81, 5.65)	1.23 (0.55, 2.75)	1.00 (0.45, 2.19)	1.00 (0.42, 2.46)
Heart disease	0.98 (0.51, 1.90)	1.79 (0.72, 4.46)	1.66 (0.72, 3.81)	0.98 (0.49, 1.99)	1.73 (0.94, 3.21)	1.37 (0.67, 2.81)
Prostate disease	1.58 (0.74, 3.37)	**2.77 (1.10, 6.92)** *	1.79 (0.68, 4.70)			

**Note:** In the odds ratios from logistic regression (and 95% confidence intervals) of these analyses, the presence of a sexual dysfunction included only those respondents who reported having experienced the problem “sometimes” or “frequently” (i.e. those who indicated “occasionally” were recorded as indicating no sexual problem). Based on reports from sexually active subjects.

* p < 0.05;

† p < 0.01;

‡ p < 0.001.

### Help-seeking behavior

The prevalence of selected help-seeking behavior for sexual problems in Brazil is summarized in Table 4. Of the respondents who were sexually active and reported experiencing at least one sexual problem, 41.3% of men and 29.7% of women had not sought any help or advice (i.e. no action taken). Patterns of help-seeking behavior showed some differences between men and women in Brazil. More than twice as many women (44.0%) as men (21.2%) reported talking to a medical doctor about their sexual problem(s). However, overall, about half of the women (53.1%) and almost three-quarters of the men (72.1%) had sought no help from a health professional. Talking to their partner was a similarly popular course of action among men and women (42.3% and 41.4%, respectively), while women were more likely than men to talk to a friend or family member (25.3% versus 10.6%) and to seek information from an anonymous source such as books and magazines, telephone help-lines or the internet (18.3% versus 12.0%). Few men (3.8%) and women (6.2%) reported seeking help from a member of the clergy or other religious adviser.

**Table 4.** Prevalence of selected help seeking behaviors for sexual problems by gender, Brazil, 2001-2002

	% (95% confidence interval)
**Men**	
Talked to partner	42.3 (35.5, 49.3)
Talked to medical doctor	21.2 (15.8, 27.3)
Taken drugs/used devices or talked to pharmacist	18.8 (13.7, 24.7)
Looked for information anonymously (in books/magazines or via telephone help-line/internet)	12.0 (7.9, 17.2)
Talked to family member/friend	10.6 (6.7, 15.6)
Talked to psychiatrist, psychologist or marriage counselor	6.7 (3.7, 11.0)
Talked to a cleric or religious adviser	3.8 (1.7, 7.4)
**Sought no help from a health professional**	**72.1 (65.5, 78.1)**
**No action taken**	**41.3 (34.6, 48.4)**
Women	
Talked to medical doctor	44.0 (38.0, 50.1)
Talked to partner	41.4 (35.5, 47.5)
Talked to family member/friend	25.3 (20.2, 30.9)
Taken drugs/used devices or talked to pharmacist	22.7 (17.9, 28.1)
Looked for information anonymously (in books/magazines or via telephone help-line/internet)	18.3 (13.9, 23.4)
Talked to psychiatrist, psychologist or marriage counselor	8.4 (5.4, 12.4)
Talked to a cleric or religious adviser	6.2 (3.7, 9.8)
**Sought no help from a health professional**	**53.1 (47.0, 59.2)**
**No action taken**	**29.7 (24.5, 35.3)**

**Note:** based on reports from respondents complaining of at least one sexual problem. All prevalences are adjusted according to the age distribution of the total of sexually active men and women in this sample from Brazil.

### Factors associated with seeking medical help for sexual problems

A number of physical, socioeconomic and attitudinal factors that might be associated with seeking medical help for sexual problems were investigated using logistic regression and the results (odds ratios) for men and women in Brazil are summarized in Table 5. Women with a high or medium household income were more likely than those with a low income to seek medical help for sexual problems (odds ratio 2.18; p < 0.05), but income was not a significant factor for men. None of the male sexual problems investigated were significantly associated with seeking medical help, but among women lubrication difficulty was a significant correlate of seeking medical help (odds ratio 2.66; p < 0.01). Having been asked by a doctor about possible sexual difficulties during a routine visit in the past three years was a significant correlate of seeking medical help for sexual problems for both men (odds ratio 4.72; p < 0.01) and women (odds ratio 1.92; p < 0.05) in Brazil. Sexual attitudes and beliefs that were significantly associated with seeking medical help for sexual problems were: among women, thinking that a doctor should routinely ask patients about sexual problems (odds ratio 2.44; p < 0.05); and among men, being somewhat or very dissatisfied with their sexual function (odds ratio 3.16; p < 0.05) and believing that sex is an extremely or very important part of life (odds ratio 2.79; p < 0.05).

**Table 5.** Factors associated with seeking medical help for sexual problems by gender, Brazil, 2001-2002

	Men	Women
**Age (years)**		
40–49	Reference	Reference
50–59	1.48 (0.51,4.27)	1.12 (0.58, 2.16)
60–69	2.18 (0.70, 6.72)	0.93 (0.41, 2.08)
70–80	2.53 (0.72, 8.94)	0.46 (0.13, 1.65)
**Education**		
Primary school or less	Reference	Reference
Secondary/high school	1.60 (0.57, 4.49)	1.46 (0.76, 2.80)
At least some college	1.74 (0.55, 5.94)	1.86 (0.83, 4.17)
High/medium household income (versus low)	1.00 (0.29, 3.51)	**2.18 (1.1, 4.72)** *
**Sexual problems**		
Erectile difficulties	1.37 (0.60, 3.16)	
Early ejaculation	0.54 (0.22, 1.30)	
Lack of sexual interest	1.17 (0.49, 2.82)	0.69 (0.38, 1.25)
Inability to reach orgasm		1.00 (0.56, 180)
Lubrication difficulties		**2.66 (1.48, 4.79)** †
**General sexual Attitudes**		
Have been asked by a doctor about possible sexual difficulties in a routine visit in the past three years	**4.72 (1.72, 12.98)** †	1.92 (1.09, 3.70)*
Think a doctor should routinely ask patients about sexual function	0.93 (0.27, 3.27)	**2.44 (1.05, 5.67)** *
Very/somewhat dissatisfied with sexual function	**3.16 (1.1,9.89)** *	1.77 (0.89, 3.54)
Belief that decreased ability to perform sexually would significantly affect self-esteem	1.10 (0.47, 2.59)	0.88 (0.49, 1.59)
Belief that sex is a extremely/very important part of overall life	**2.79 (1.10, 7.11)** *	0.91 (0.30, 2.78)
Think it is OK to use medical treatment for sexual problems	1.08 (0.35, 3.33)	0.71 (0.31, 1.63)
Think that older people no longer want/have sex	1.12 (0.48, 2.65)	0.61 (0.34, 1.11)
Belief in religion guiding sex	0.79 (0.33, 1.90)	1.06 (0.60, 1.86)

**Note:** odds ratios from logistic regression (and 95% confidence intervals). Based on reports from respondents complaining of at least one sexual problem.

* p < 0.05;

† p < 0.01

### Attitudes and beliefs about diagnosis and treatment of sexual problems

By far the most common reason cited by men and women in Brazil for not consulting a doctor about a sexual problem was a belief that it is a normal part of aging, or being comfortable as he/she is (81.7% of men and 85.6% of women) (Table 6). However, thinking the problem was not very serious or waiting for it to go away (59.1% of men and 69.9% of women); feelings of discomfort or embarrassment about talking to a doctor (52.4% of men and 60.8% of women); and thinking it was not a medical problem or that a doctor could not do much to help (57.9% of men and 58.8% of women), were all cited by more than half of all respondents (Table 6). Lack of access to or affordability of medical care was also cited as a reason by about half of the men (54.9%) and women (46.4%). Few respondents in Brazil had been asked by a doctor about possible sexual difficulties during a routine visit in the past 3 years (13.6% of men and 19.8% of women) but more than three-quarters of men (85.4%) and women (76.9%) thought that a doctor should routinely ask patients about their sexual function.

**Table 6.** Attitudes, behaviors and beliefs about diagnosis of and treatment for sexual problem by gender, Brazil, 2001-2002

	% (95% confidence interval)
**Men**	
**Reasons for not consulting a doctor about the sexual problem experienced:** *	
Normal with aging/I am comfortable the way I am	81.7 (74.9, 87.3)
Did not think it was very serious/Waiting to see if problem goes away	59.1 (51.2, 66.7)
Doctor cannot do much/Do not think it is a medical problem	57.9 (50.0, 65.6)
Do not have a regular physician/Doctor is expensive	54.9 (46.9, 62.6)
Not comfortable talking to a MD/MD is a close friend/MD is the wrong gender	52.4 (44.5, 60.3)
Doctor uneasy to talk about sex	25.6 (19.1, 33.0)
Have been asked by a doctor about possible sexual difficulties in a routine visit in the past three years†	13.6 (10.6, 17.0)
Think a doctor should routinely ask patients about their sexual function†	85.4 (81.8, 88.4)
**Women**	
**Reasons for not consulting a doctor about the sexual problem experienced:** *	
Normal with aging/I am comfortable the way I am	85.6 (79.0, 90.8)
Did not think it was very serious/Waiting to see if problem goes away	69.9 (62.0, 77.0)
Not comfortable talking to a MD/MD is a close friend/MD is the wrong gender	60.8 (52.6, 68.6)
Doctor cannot do much/Do not think it is a medical problem	58.8 (50.6, 66.7)
Do not have a regular physician/Doctor is expensive	46.4 (38.3, 54.6)
Doctor uneasy to talk about sex	22.2 (15.9, 29.6)
Have been asked by a doctor about possible sexual difficulties in a routine visit in the past three years†	19.8 (16.9, 22.9)
Think a doctor should routinely ask patients about their sexual function†	76.9 (73.7, 79.9)

* Based on reports from respondents complaining of at least one sexual problem who have not consulted a doctor.

† Based on all respondents. All prevalences are adjusted according to the age distribution of the total of sexually active men and women in this sample from Brazil. MD = medical doctor.

## DISCUSSION

The Global Study of Sexual Attitudes and Behaviors has obtained population-level data on sexual attitudes and behaviors from middle-aged and older adults in 29 countries in a manner that allows direct comparisons of the results from different countries and regions. The large, cross-national sample and the use of a common method of data collection represent two major strengths of this study. Here, we have focused specifically on the sexual activity, prevalence of sexual problems and associated help-seeking behavior among men and women in Brazil. The standardized, structured questionnaire was administered using computer-assisted telephone interviews. This method was chosen in preference to face-to-face interviews to avoid causing respondents undue embarrassment when talking about private and sensitive issues, and to minimize the likelihood that they might feel obliged to give “socially desirable” answers.^26^

Only sexual problems that were experienced periodically or frequently (i.e. those that persisted with moderate to higher frequency) were considered to be “dysfunctions”.^27^ This is essentially equivalent to using two sequential screening tests, and minimizes the risk of false positive responses. It is likely, therefore, that the prevalence of sexual dysfunction may be under-reported in the Global Study of Sexual Attitudes and Behaviors, in comparison with studies that used more sensitive, but less specific, methods. Published studies on the prevalence of male erectile dysfunction in Brazil have highlighted the need to consider the severity or frequency of a sexual dysfunction when comparing reports from apparently similar study samples. Two population-based surveys conducted in Brazil, one in the northeast^13^ and the other in the southeast,^14^ reported a prevalence of moderate or complete erectile dysfunction of 14.4% and 12.0%, respectively, among Brazilian men aged 40 to 70 years. Likewise, in a national survey in Brazil, the prevalence of moderate or complete erectile dysfunction was 14.7%.^12^ These estimates are similar to what was observed in the GSSAB Brazilian cohort, in which the overall age-standardized prevalence of erectile difficulties was 13.8% (9.8% experienced the problem frequently or periodically).

The overall response rate in Brazil (18.4%) was low, but the prevalence of a number of self-reported health conditions, including hypertension, diabetes and smoking in GSSAB was comparable with published values.^28–31^ This suggests that refusal to participate in this study was most probably due simply to an unwillingness to undergo a telephone interview and the modest response rate is therefore unlikely to have introduced a bias in the estimates of the prevalence of sexual behaviors and problems. It also appears to indicate that the study population was broadly representative of the Brazilian population. This assumption is further supported by the observation that the prevalence of erectile difficulties among men in the Brazilian cohort of the GSSAB was comparable to what has been reported in published studies, which have focused on the prevalence of moderate or severe erectile dys-function among Brazilian men aged between 40 and 70 years.^13,14,32^

A diagnosis of depression was a significant correlate of a lack of sexual interest for both genders, and of erectile difficulties and early ejaculation among men in the GSSAB Brazilian cohort. A lack of sexual interest was also significantly associated with prostate disease and early ejaculation with a diagnosis of hypertension. Interestingly, none of the reported medical conditions, apart from depression, were significant correlates of sexual problems among Brazilian women in the GSSAB. Comorbidity between erectile dysfunction and depression is known to exist but the precise nature of the relationship between these conditions is not clear.^33^ While it is possible that the distress of erectile dysfunction may contribute to the development of depressive illness, it is also possible that depressive illness may lead to erectile difficulties. Moreover, it is important that the possible role of antidepressant treatments is considered when investigating the copresence of depression and sexual dysfunction. Sexual dysfunction is a well-recognized side effect of antidepressant therapy. However, different agents may be associated with different rates of dysfunction.^34–36^ Selective serotonin reuptake inhibitors (SSRIs) have been reported to be associated with particularly high rates of sexual dysfunction and, while men report higher rates of sexual side effects from SSRIs than do women, women seem to experience more severe dysfunction with these agents.^35,37^ A significant association between depression and erectile dysfunction among men in Brazil has been reported previously.^12–14^ However, the data from GSSAB indicate that depression is also associated with other male sexual problems, namely early ejaculation and a lack of sexual interest. Despite the correlation between sexual problems and depression, the relationship is most probably bi-directional, i.e. sexual problems may follow depression, while depression may be a consequence of sexual dysfunction. We cannot discern the causal direction in these cross-sectional data. The association between depression and sexual dysfunction warrants further investigation because depression is highly prevalent in Brazil and other Latin American countries, possibly due at least in part to social factors such as violence that are especially present in medium and large cities.^38^ (For logistical reasons, the Brazilian sample in GSSAB was drawn primarily from urban areas.)

The GSSAB data indicate that feeling that the problem is not severe, or not being bothered by the problem, may be deterring men and women in Brazil from discussing their sexual difficulties with their doctor. Furthermore, it appears that doctors in Brazil rarely ask patients about their sexual health during a routine consultation, even though the vast majority of men and women would appreciate this and it would appear to encourage medical help-seeking for sexual problems. Untreated sexual problems can greatly impair a patient's enjoyment of their sexual life and it is important that physicians, especially primary care physicians, ask patients about possible sexual difficulties during routine visits.^39^ This should result in improved sexual functioning for the patient and an enhanced physician-patient relationship and greater professional satisfaction.

Socioeconomic factors also influence patterns of help-seeking behavior for sexual problems among mature men and women in Brazil. Our findings indicate that women with a medium or high household income were significantly more likely to seek help than women from low-income households. Furthermore, a lack of access to or affordability of medical care was cited by about one-half of all respondents, both men and women, as a reason for not seeking medical help for sexual problems. A recent cross-sectional study performed in Rio Grande do Sul, Brazil, showed that only 37% of the study sample had a regular doctor and that this was directly associated with income.^40^ The authors also found that individuals with a regular physician tended to have better access to a range of health services and they recommended encouraging people to consult with a regular doctor as a means of improving the quality of and access to healthcare services, particularly among the poorest individuals. Improving the care available to older adults with low income may be especially important, as a study of the influence of socioeconomic circumstances on health has demonstrated that, among a representative sample of the Brazilian population aged 65 years or older, lower income was associated with worse health and physical functioning, and less frequent use of medical services.^41^

## CONCLUSIONS

We conclude that, although middle-aged and elderly men and women in Brazil continue to show sexual interest and activity, a number of sexual problems are highly prevalent. Only a minority of the men and women who experience sexual difficulties seek medical help: this may be partly because they do not perceive such problems as potentially treatable medical conditions, or because they do not have access to or cannot afford medical care. The findings from GSSAB highlight the importance of encouraging greater use of the available healthcare services, including consultation with a medical doctor on matters of sexual health. This should not only enable men and women to maintain satisfactory sexual function well into their later years, but may also result in an overall improvement in the quality of healthcare, particularly among poorer individuals.
